# Chemical-genomic profiling identifies genes that protect yeast from aluminium, gallium, and indium toxicity

**DOI:** 10.1093/mtomcs/mfad032

**Published:** 2023-05-16

**Authors:** Yves Schulze, Payam Ghiaci, Liqian Zhao, Marc Biver, Jonas Warringer, Montserrat Filella, Markus J Tamás

**Affiliations:** Department of Chemistry and Molecular Biology, University of Gothenburg, S-405 30 Göteborg, Sweden; Department F.-A. Forel, University of Geneva, Boulevard Carl-Vogt 66, CH-1205 Geneva, Switzerland; Department of Chemistry and Molecular Biology, University of Gothenburg, S-405 30 Göteborg, Sweden; Department of Chemistry and Molecular Biology, University of Gothenburg, S-405 30 Göteborg, Sweden; Bibliothèque Nationale du Luxembourg, 37D Avenue John F. Kennedy, L-1855 Luxembourg, Luxembourg; Department of Chemistry and Molecular Biology, University of Gothenburg, S-405 30 Göteborg, Sweden; Department F.-A. Forel, University of Geneva, Boulevard Carl-Vogt 66, CH-1205 Geneva, Switzerland; Department of Chemistry and Molecular Biology, University of Gothenburg, S-405 30 Göteborg, Sweden

**Keywords:** aluminium, gallium, indium, toxicity, yeast, chemical-genomic profiling

## Abstract

Aluminium, gallium, and indium are group 13 metals with similar chemical and physical properties. While aluminium is one of the most abundant elements in the Earth's crust, gallium and indium are present only in trace amounts. However, the increased use of the latter metals in novel technologies may result in increased human and environmental exposure. There is mounting evidence that these metals are toxic, but the underlying mechanisms remain poorly understood. Likewise, little is known about how cells protect themselves from these metals. Aluminium, gallium, and indium are relatively insoluble at neutral pH, and here we show that they precipitate in yeast culture medium at acidic pH as metal-phosphate species. Despite this, the dissolved metal concentrations are sufficient to induce toxicity in the yeast *Saccharomyces cerevisiae*. By chemical-genomic profiling of the *S. cerevisiae* gene deletion collection, we identified genes that maintain growth in the presence of the three metals. We found both shared and metal-specific genes that confer resistance. The shared gene products included functions related to calcium metabolism and Ire1/Hac1-mediated protection. Metal-specific gene products included functions in vesicle-mediated transport and autophagy for aluminium, protein folding and phospholipid metabolism for gallium, and chorismate metabolic processes for indium. Many of the identified yeast genes have human orthologues involved in disease processes. Thus, similar protective mechanisms may act in yeast and humans. The protective functions identified in this study provide a basis for further investigations into toxicity and resistance mechanisms in yeast, plants, and humans.

## Graphical Abstract

**Graphical Abstract:**
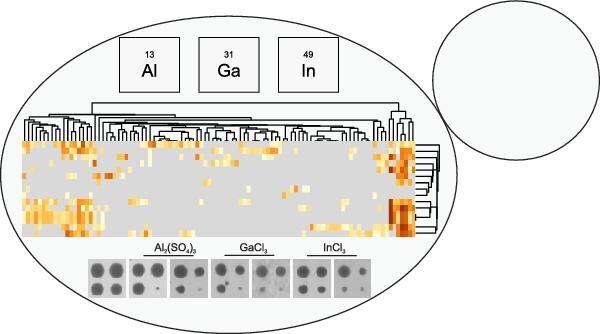
Yeast defence against aluminium, gallium, and indium toxicity.

## Introduction

Cells and living organisms depend on a range of metals for numerous biological processes, some of which are essential for life. Many other metals have no known biological role and are highly toxic already at very low concentrations inside cells. Therefore, cells have evolved molecular systems that distinguish essential or beneficial metals from the harmful ones.^1–3^ Metals are present in the environment in a vast concentration range with aluminium (Al) being one of the most abundant elements in the Earth's crust, whereas most other metals, such as gallium (Ga) and indium (In), are present in trace amounts.^4,5^ Al has been used in technological applications for more than a century, e.g. in the construction industry, in food preparation and storage, and as adjuvant in vaccines.^6^ Thus, microbes, plants, and humans are continuously exposed to Al from both natural and anthropogenic sources. In contrast, exposure to trace metals such as Ga and In is generally expected to be low, but their accelerating use in modern technologies has raised concerns for increased human and environmental exposure.^7,8^ Ga and In are important components in novel technology applications such as in electronics, semiconductor, and energy technologies,^9,10^ and In- and Ga-containing compounds are used as anti-tumour and antimicrobial agents.^1,7,11–13^

Al, Ga, and In have no known biological roles and have been shown to be toxic to plants^14–18^ and microbes,^1^ and to have adverse health effects on humans and experimental animals.^6,7,11,12^ In exposure in occupational settings can damage the liver and spleen, and result in lung disease and renal failure in humans.^7,12,19^ On the cellular level, In appears to target the endoplasmic reticulum (ER) and haem metabolism.^7,12,20^ Ga-containing compounds can affect the lungs, the kidney, and the immune and hematopoietic systems in experimental animals.^11^ At the cellular level, Ga causes increased levels of reactive oxygen species, perturbs iron (Fe) homeostasis and Fe-dependent processes, and inhibits ribonucleotide reductase, a key enzyme in DNA synthesis.^11,13^ In humans, Al has been shown to affect the lungs, the cardiovascular system, the central nervous system, and the bone. At the cellular level, exposure to Al has been associated with oxidative stress, genotoxicity, disruption of mineral metabolism, membrane perturbation, and enzymatic dysfunction.^6,21^ The presence of Al in acidic soils limits plant growth and crop yield, and Al toxicity for plants has been attributed to its interference with the absorption of water and mineral nutrients such as phosphorus (P), potassium (K), calcium (Ca), magnesium (Mg), Fe, molybdenum (Mo), and boron (B).^14,15^ Similarly, Ga and In appear to affect nutrient levels in rice seedlings.^22^ Nevertheless, the molecular mechanisms underlying Al, Ga, and In toxicity are not well understood. Likewise, little is known about how cells protect themselves from the toxic effects caused by these metals.

The toxicity of a metal depends on its chemical properties, its oxidation states, the complexes formed in the intra- and extracellular environment, and its interactions with cellular macromolecules such as DNA and proteins.^1,3,23^ Al, Ga, and In are group 13 metals and have similar chemical and physical properties: They primarily exist as trivalent elements in natural systems, they have a relatively high charge and small radii, and they behave as hard acids preferentially binding to oxygen-, less to nitrogen-, and least to sulphur-containing ligands.^24,25^ In, because of its larger ionic radius, has a slightly higher affinity for softer ligands than Al and Ga. In and Ga form covalent bonds more easily than Al because they carry full d-orbitals.^24^ In and Ga also share some properties with Fe(III), including ionization potential, ionic radii, and coordination number of their aquo 3+ cations, and they may replace Fe(III) in important cellular functions.^26^

A number of Al detoxification systems have been described in plants.^14,27^ In contrast, very little is known about how cells protect themselves from the toxic effects of Ga and In. Although exposure to Ga and In is low in nature, their abundance is comparable to that of arsenic (As), Mo, iodine (I), and selenium (Se), for which cells have evolved dedicated detoxification or utilization systems.^28,29^ Thus, cells might possess systems that confer resistance to Ga and In. The budding yeast *Saccharomyces cerevisiae* is a powerful eukaryotic model system to elucidate how metals and other compounds affect molecular processes and by which means cells defend themselves.^30–32^ The knowledge gained from studies in yeast may also be relevant from a human health perspective, since metal toxicity and defence mechanisms are often evolutionarily conserved. Thus, studies in yeast may suggest routes for targeted experimentation and treatment in higher models such as plants and humans.^32^

In this study, we screened the *S. cerevisiae* single-gene deletion collection consisting of ∼4300 knockout strains lacking individual non-essential genes, and identified genes and biological processes required for cell proliferation in the presence of Al, Ga, and In. Our analyses pinpointed shared and metal-specific genes and biological processes that confer resistance to these metals. Several of the identified gene products have human orthologues, suggesting that similar protective mechanisms may act also in humans.

## Methods

### Chemicals

Aluminum sulphate octadecahydrate Al_2_(SO_4_)_3_ · 18H_2_O, purity 99.4% (BDH Laboratory), indium chloride InCl_3_, purity 98% (Sigma–Aldrich), and gallium chloride GaCl_3_, purity 99.999% (Indium Corporation) were used.

### Yeast strains, plasmids, and cultivation media

The yeast strains used in this study include the haploid single-gene deletion collection (BY4741 background: *MAT***a**  *his3Δ1 leu2Δ0 met15Δ0 ura3Δ0*) and the homozygous diploid deletion collection (BY4743 background: *MAT****a****/α his3Δ1/his3Δ1 leu2Δ0/leu2Δ0 LYS2/lys2Δ0 met15Δ0/MET15 ura3Δ0/ura3Δ0*).^33^ Yeast cells were stored at −80°C in 20% glycerol and cultivated at 30°C. The size of the BY4743 deletion collection was 4308 strains, after leaving out 287 (6.7%) strains with very slow growth on the background medium (without metal) for which a reliable estimate of metal sensitivity could not be obtained. Strains were cultivated on rich Yeast Peptone Dextrose (YPD) medium with 2.0% (w/w) glucose, or on synthetic complete (SC) medium composed of 0.14% Yeast Nitrogen Base (YNB; CYN2210, ForMedium), 0.50% (NH_4_)_2_SO_4_, 0.077% Complete Supplement Mixture (CSM; DCS0019, ForMedium), and 2.0% (w/w) glucose. pH was set to 4.3. For all solid medium cultivations, 2.0% (w/v) agar was added in the absence or presence of Al_2_(SO_4_)_3_, InCl_3_, or GaCl_3_ added from 100 mM stock solutions to warm (60–80°C) medium. Solid medium plates were cast 10–15 h prior to use in PlusPlates (Singer Instruments, UK), on a levelled surface, by pouring 50 ml of medium in the upper right corner of each plate. Excess liquid was removed by drying the plates in a laminar airflow in a sterile environment. No contamination was observed on any plate. The plasmid containing *IRE1* behind its own promoter in pRS313 has been described previously.^34^

### Cell cultivation

Cell transfers between storage and pre-cultivation experimental plates were done using robotics (ROTOR HDA, Singer Instruments Ltd, UK) at the indicated transfer format. First, we thawed and resuspended frozen stocks (20% glycerol), transferred a sample (96 long pin pads) to a solid YPD medium, and pre-cultivated the transferred cells as colonies until stationary phase (72 h). We then sampled and transferred cells (∼10^5^ per colony) from the YPD pre-culture colonies (1536 short pin pads) to solid background medium target plates to generate a 1152 colony array of experimental (gene deletion) strains. In every fourth position, we also interleaved (384 short pin pad) *his3Δ* control colonies (*n* = 384 on each plate) carrying the same *KANMX* deletion marker as the experimental colonies, which had been pre-cultivated on a separate YPD source plate and subsampled. Because *HIS3* is turned off in the presence of external histidine, its absence should have no effect on colony growth. We then cultivated the 1152 experimental colonies and 384 control colonies on shared plates in a second pre-culture. Finally, we transferred (1536 short pin pads) cells sampled from the 1536 pre-culture colonies to experimental plates containing SC or YPD medium, with and without metal added. We assayed the growth of these experimental cell populations for 72 h using the Scan-o-Matic system^35^ version 1.5.7 (https://github.com/Scan-o-Matic/scanomatic.git), which measures the amount of light transmitted through each colony and estimates the number of yeast cells present. The experimental plates were maintained undisturbed and without lids for the duration of the experiment (72 h) in high-quality desktop scanners (Epson Perfection V800 PHOTO scanners, Epson Corporation, UK) standing inside dark, temperature-controlled (30°C) and moisture-controlled thermostatic cabinets with air circulation. We imaged plates at 20 min intervals using transmissive scanning at 600 dpi, identified the position of colonies, and recorded the transmitted light intensities for pixels included in, and outside, each colony. For each colony, we estimated its sum pixel intensity as well as the median pixel intensity of pixels defined as belonging to the local background. We subtracted the latter from the former and converted the remaining cell-associated pixel intensity to cell counts by using a pre-established calibration function, which had been obtained from previous experiments by comparing Scan-o-Matic estimated cell numbers to those obtained using both spectrometry and flow cytometry. We smoothed and quality controlled growth curves, rejecting ∼0.3% of growth curves as erroneous while being blinded to sample identities (for details, see Zackrisson et al.^35^).

### Cell doubling-time extraction

We extracted the cell doubling time, *D*, from expanding cell populations. We used the 384 fixed spatial controls introduced at every fourth position to account for systematic doubling-time variations within and across plates. By interpolating across the log_2_(*D*) values of the 384 measured controls (see Zackrisson et al.^35^), we estimated the log_2_(*D*) value a control colony would have had in each position. From the log_2_(*D*) value for each colony, we then subtracted the corresponding log_2_(*D*) control value, thereby obtaining a normalized, relative log_2_ doubling time, log_2_(*D*)*_norm_*. The growth of some deletion strains deviates from that of the *his3Δ* control already in the background medium, i.e. they have a growth aberration that is not caused by a metal but by the background medium. To account for this, we estimated the specific impact of each metal on the doubling time of each gene deletion strain to obtain a Logarithmic Phenotypic Index, *LPI_stressor_. LPI_stressor_* = log_2_(*D*)*_norm, stressor—_*log_2_(*D*)*_norm, background medium_*, where log_2_(*D*)*_norm, stressor_* and log_2_(*D*)*_norm, background medium_* are the mean relative growths on metal and background medium, respectively, across replicates (*n* = 3). A positive LPI corresponds to the gene deletion strain being more sensitive than the *his3Δ* control to the specific stressor/metal in question, while a negative LPI corresponds to the gene deletion strain being more resistant than the control.

### Statistics

We called significant, metal-specific differences in growth between each deletion strain and the *his3Δ* control as significant deviations in *LPI_stressor_* from 0. We used a one-sample *t*-test and corrected for the multiple hypothesis tested using a Benjamini–Hochberg False Discovery, with a significance threshold of 1% expected false positives (α = 0.01). In addition, to account for the expected low variation of some strain due to chance measurement effects, we also required that a significant LPI deviation from 0 should be very strong (LPI ≥ 2.5). This corresponds to a mutant being 5.6-fold more sensitive to a specific metal than the wild type, in terms of the cell doubling time.

### Bioinformatics and network analyses

Gene Ontology (GO) enrichment analysis was performed with the Metascape database^36^ with the following settings: *P *< 0.01, minimal overlap genes = 3, and minimal enrichment factor > 1.5. The *P*-values were Benjamini–Hochberg corrected. Protein–protein interaction networks were constructed using the STRING database^37,38^ with the organism set to *S. cerevisiae* and the confidence score to high (0.700). The functional diversity of the gene lists was also visualized by mapping each hit onto the global yeast genetic interaction network using CellMap.^39,40^ Putative Human orthologues of the identified yeast genes and their associated OMIM^41^ disease phenotypes were retrieved from the SGD YeastMine platform (https://yeastmine.yeastgenome.org).

### Chemical speciation calculations

The chemical speciation of Al, Ga, and In in the yeast synthetic complete culture medium was calculated with the JESS software (http://jess.murdoch.edu.au/jess_home.htm) by using the current default database. This database had recently been updated for Ga and In after performing an extensive search of literature values. This comprehensive search showed that equilibrium constants for many equilibria were missing and that some of the existing ones were doubtful, which is unfortunately common for many less-studied elements. Despite these limitations, exploratory calculations were run because of their intrinsic interest. Equilibrium constant values used for the calculations are accessible at the JESS webpage. Only the inorganic components were considered because too few equilibrium constants of equilibria involving the organic ligands present in the culture medium were available (Table S1).

### SEM–EDXS analysis

Metal precipitates were filtered, dried at 100°C, observed by scanning electron microscopy (SEM), and their chemical composition measured by energy-dispersive X-ray spectroscopy (EDXS). Samples were mounted on aluminium stubs using double-sided conductive carbon tape and coated with Au (ca. 10 nm) using low vacuum sputter coating. A JEOL JSM-7001F scanning electron microscope, equipped with an EDXS detector (model JEOL EX- 94300S4L1Q), was used to perform analyses. EDXS measurements were acquired with an accelerating voltage of 15 kV, a beam current of 7 nA, and acquisition times of 30 s. Semiquantitative EDXS analyses of elemental concentrations were made without taking C, N, and O into account (elemental quantification is not as good for light elements). EDXS detection limits are dependent on several parameters but estimated to be around 1% (weight).

## Results and discussion

### Al, Ga, and In precipitate in the yeast culture medium

Metal solubility is an important issue to consider when performing standardized and reproducible toxicity assays because it can affect the true, dissolved concentration of the element. Al, Ga, and In are relatively insoluble at neutral pH but the solubility of Al-, Ga-, and In-hydroxides is known to increase when the pH becomes more acidic.^42,43^ In fact, the increased solubility of Al has been proposed to be the origin of Al toxicity to fishes in lakes affected by acid rain^42,43^ and to plants growing on acidic soils.^14,15^ In this work, we chose an acidic pH (4.3) for all yeast experiments to enhance solubility of these metals.

The presence of complexing ligands can affect metal solubility. We calculated the chemical speciation of Al, Ga, and In in the yeast synthetic complete culture medium using the JESS software. Only the inorganic components were considered because too few equilibrium constants of equilibria involving the organic ligands present in the yeast synthetic complete culture medium were available (Table S1). The calculations predicted supersaturation of insoluble species for the three elements, mostly as phosphates (Table S2). ‘Dissolved’ species were phosphate-containing ones, except in the case of In, but this is due to the lack of any constant for such species in the database. As expected, no free metal ion is predicted to be present in significant amounts in the medium and even the hydrolyzed species usually predominant for these elements at pH 4.3 are nearly not formed in the presence of the high concentration of phosphate in the yeast culture medium. Since the charge of the chemical species is known to play a role in metal bioavailability, it is to be mentioned that all predicted ‘dissolved’ Al-containing species were neutral at the nominal pH of the experiments. A better characterization of Ga- and In-phosphate systems would be needed to ascertain whether this is also the case for these elements.

**Fig. 1:**
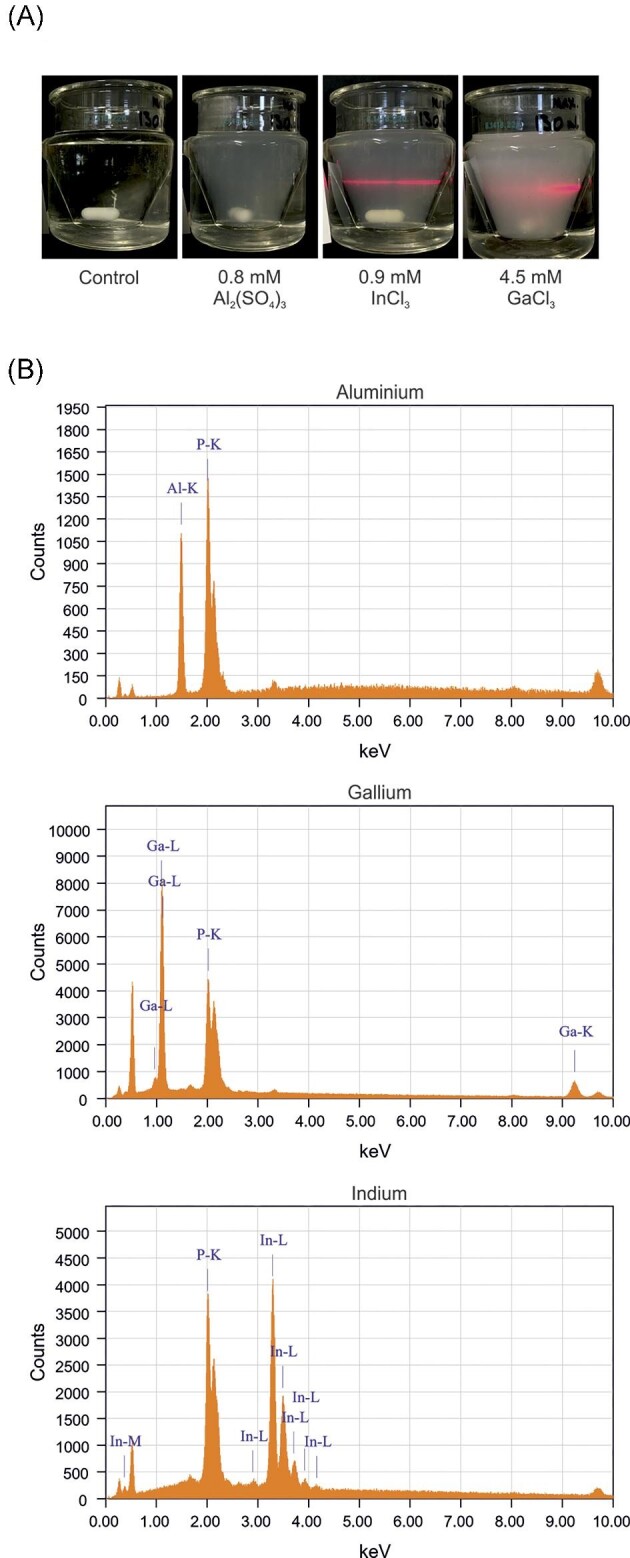
Al, Ga, and In precipitate in the yeast culture medium. (**A)** Turbidity of yeast synthetic minimal culture medium without (control) or with metal added at the indicated concentrations and pH 4.3. The Tyndall effect observed by using a laser beam corroborates the formation of a solid phase in all cases. (**B)** EDXS spectra of the precipitates formed in the suspensions shown in panel A.

The formation of insoluble species in the yeast synthetic complete culture medium was then assessed by preparing mixtures of each element using concentrations (1.6 mM Al, 0.9 mM In, and 4.5 mM Ga) that have only minor effects on yeast proliferation (Fig. S1A). Turbidity appeared in all cases and the Tyndall effect observed using a laser beam corroborated the formation of solid phases in all cases (Fig. 1A). Thus, all three metals precipitate in the yeast synthetic complete culture medium already at marginally stressful concentrations.

The precipitates were collected by filtration and observed by SEM, and their chemical composition measured by EDXS. The presence of phosphate was observed in all cases (Fig. 1B). The following molar ratios were obtained: P/Al: 1.4, P/Ga: 1.0, and P/In: 0.6. Only in one case (for Ga), the molar ratio seems to confirm the formation of a solid with the expected stoichiometry according to the calculations. However, note that the solution chemistry of these elements is not straightforward. For instance, in the case of Al, the most studied element of the three considered in this study, hydroxy-Al polymers are formed that yield different reaction products with phosphate depending on the Al:P ratio.^44^ Similar behaviour could be expected for Ga and In. Nevertheless, the finding that the precipitates consisted of metal-phosphate ligands is in accordance with speciation calculations (Table S2), and with the fact that these metals are hard acids that preferentially interact with hard bases, such as phosphate.^1,24,25^

We conclude that the total dissolved concentration of Al, Ga, and In is likely to be substantially lower than the nominal one in the yeast culture medium due to the formation of insoluble species. Nevertheless, since growth of yeast cells is affected by the presence of these metals, the active concentration of soluble metal, albeit unknown, is sufficient to induce toxicity. Alternatively, we cannot exclude the possibility that these metals enter and affect cells in form of metal-ligand complexes. Although important to explain toxicity mechanisms, to define metal concentrations and the chemical species formed inside cells remains challenging. Likewise, the targets through which most metals exert their toxicity are poorly understood.

### Chemical-genomic profiling identified genes required for growth in the presence of Al, Ga, and In

**Fig. 2:**
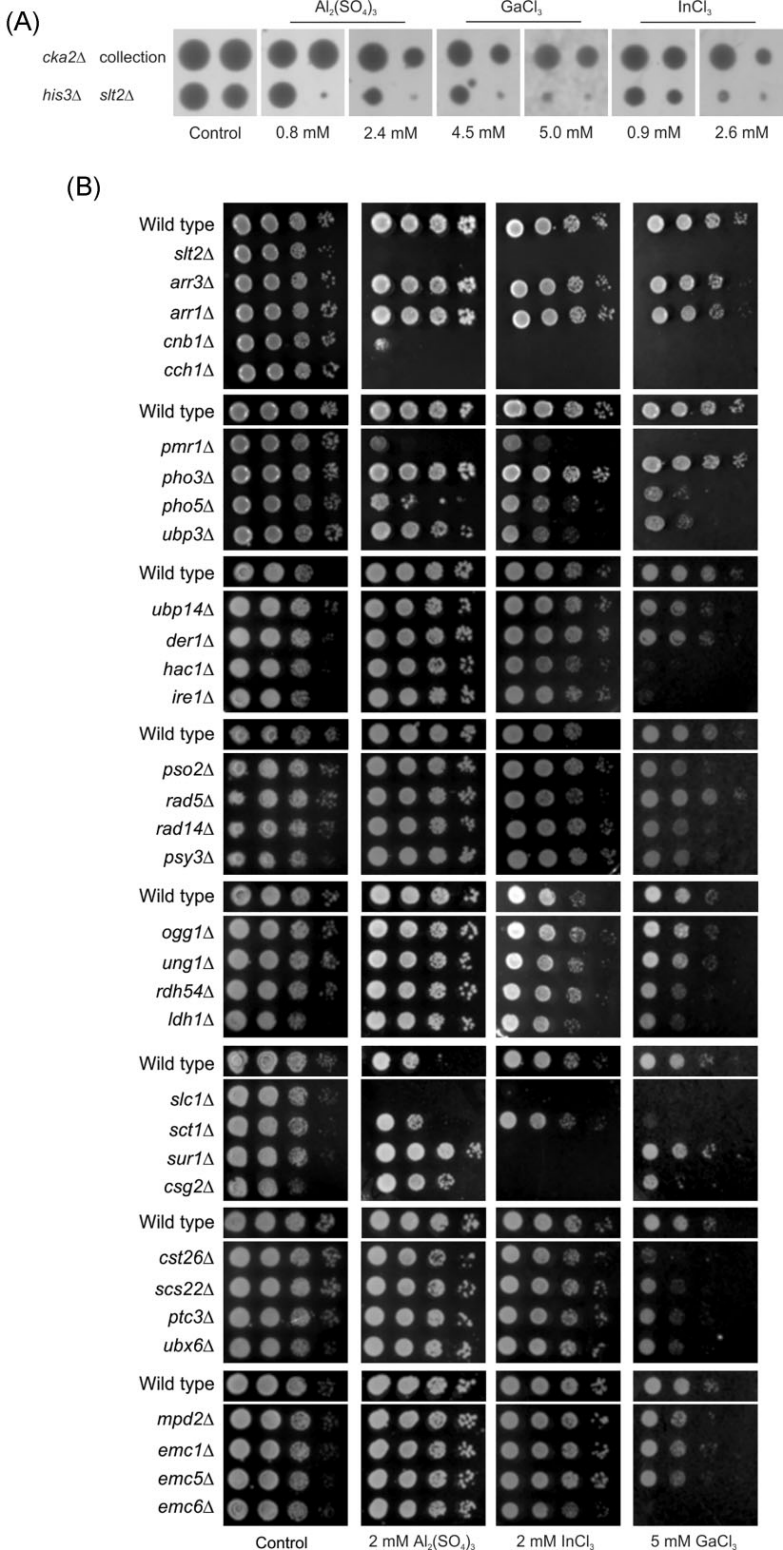
The presence of Al, Ga, and In affects yeast growth. (**A)** Preliminary automated growth tests. The indicated strains (*cka2Δ*, collection, *his3Δ, slt2Δ*) were pinned onto agar plates in the absence (control) and the presence of the metal salts at the indicated concentrations. The plates were incubated at 30°C for 3 days. Representative images are shown from at least two independent experiments. (**B)** The indicated wild type and mutant strains were pre-cultured in synthetic minimal medium, and 10-fold serial dilutions starting with an optical density (OD) at 600 nm of 1 were placed on synthetic minimal medium plates containing the indicated concentrations of metal salts. The plates were incubated at 30°C for 3 days. Representative images are shown from at least two independent experiments.

Chemical-genomic profiling using yeast *S. cerevisiae* is a powerful approach to uncover the toxic actions of metals as well as protective mechanisms.^30,31^ To set the conditions for a genome-wide chemical-genomic screen, we initially grew yeast cells on agar plates with various concentrations of Al_2_(SO_4_)_3_, InCl_3_, and GaCl_3_ ranging from 0 to 10 mM (Fig. S1A). Next, the exposure conditions and metal concentrations were adjusted to an automated high-throughput setting. We used *his3Δ* cells as the ‘wild type’ to account for potential effects of the *KANMX* deletion marker, and the mutants *slt2Δ* and *cka2Δ* that were previously shown to be, respectively, sensitive and resistant to Al, Ga, and In^45,46^ as positive controls. The automated tests showed that, using synthetic complete medium with agar at pH 4.3 and with metals at the indicated concentrations, growth of ‘wild type’ control cells (*his3Δ*) was moderately affected, permitting the identification of both metal resistant (exemplified by the larger growth area of *cka2Δ* compared with that of *his3Δ*) and metal sensitive (exemplified by the smaller growth area of *slt2Δ* compared with that of *his3Δ*) mutants (Fig. 2A). We next screened the *S. cerevisiae* homozygous diploid deletion strain (BY4743) collection consisting of 4308 mutants in total on plates containing Al_2_(SO_4_)_3_ (0.8 mM and 2.4 mM), GaCl_3_ (4.5 mM and 5.0 mM), and InCl_3_ (0.9 mM and 2.6 mM) using the Scan-o-Matic platform of continuous growth monitoring.^35^ The use of homozygous strains prevents recessive loss-of-function mutations emerging in a heterozygote state during strain construction and propagation to affect metal resistance. Higher metal concentrations resulted in longer population doubling times; however, population doubling times at the lower metal concentrations were only marginally affected (Fig. S1B). Therefore, we only considered the higher concentration for each metal (2.4 mM Al_2_(SO_4_)_3_, 5.0 mM GaCl_3_, and 2.6 mM InCl_3_) for further analysis.

We called sensitive and resistant deletion mutants using conservative thresholds (see *Methods* section for details) and, after removing dubious ORFs, obtained 171 (for Al), 133 (for Ga) and 248 (for In) metal sensitive mutants (Table S3) but only a handful of metal resistant mutants (3 for Al; 10 for Ga, 4 for In). Nearly all of the resistant mutants grew poorly in the absence of metal and were likely called resistant because the genes are required for fast growth (on medium without stress) but less during slower growth (on metal medium). They were therefore not analysed further.

To test the fidelity of the calls, we repeated the experiment for a subset of mutants called metal sensitive but using a separate collection of single-gene deletion mutants in the haploid BY4741 strain background. We confirmed the sensitivities for 8 out of 20 tested mutants for Al, 18 out of 19 for Ga, and 15 out of 26 for In (Fig. 2B). This suggests an overall true positive rate of 40% for Al, 95% for Ga, and 58% for In. Although the reason for the lower confirmation rate for Al and In sensitive mutants is unknown, we speculate that metal precipitation could be a major part of this problem. For example, slight alterations in culture media composition from one batch to another might impact metal solubility, which in turn can have a negative impact on experiment reproducibility. Furthermore, we used the homozygous diploid BY4743 for the screen, whereas we performed the validation assays with the haploid BY4741, and ploidy is known to have substantial environment-specific effects on yeast growth.^47^ Finally, the screen identified sensitive mutants based on their (diminished) growth rate (i.e. relative cell doubling time), while the validation assay scored for a combination of lag phase, growth rate, and survival. The confirmation rate obtained here using dissimilar screening and validation methods is comparable to the overlap (on the gene level) between chemical-genetic screens that used dissimilar screening and identification methods.^48^ Nevertheless, the true positives are probably of central importance for conferring resistance as they were verified by independent assays and in different strain backgrounds.

Since the metals form precipitates with phosphate (Fig. 1B), we were concerned that the observed phenotypes might be a result of phosphate limitation. To address this, we grew the same set of mutants on low-phosphate medium. However, none of the tested mutants showed an obvious growth defect in low-phosphate medium (Fig. S2A). We also compared our hits to a set of 68 mutants that have low intracellular phosphate levels when grown in rich YPD culture medium.^49^ The overlap was, at the best, poor between this set of mutants and the sets of genes that conferred resistance to Al (6 genes, *P *= 0.03), Ga (3 genes, *P *= 0.20), and In (3 genes, *P *= 0.20) (Fig. S2B). Thus, phosphate depletion is probably not a major contributor to the growth defects of the identified metal sensitive mutants. We also compared our hits to a set of 129 mutants that are sensitive to intracellular acid stress.^50^ Again, the overlap between this set of mutants and the sets of genes that conferred metal resistance (Al: 3 genes, *P *= 0.12; Ga: 1 gene, *P *= 0.07; and In: 5 genes, *P *= 0.11) was insignificant (Fig. S2C). Taken together, our results suggest that it is the presence of Al, Ga, and In in the culture medium that cause the growth defect of the called mutants.

### Functional analyses pinpoint shared and distinct biological processes that protect yeast cells from Al, Ga, and In toxicity

**Fig. 3:**
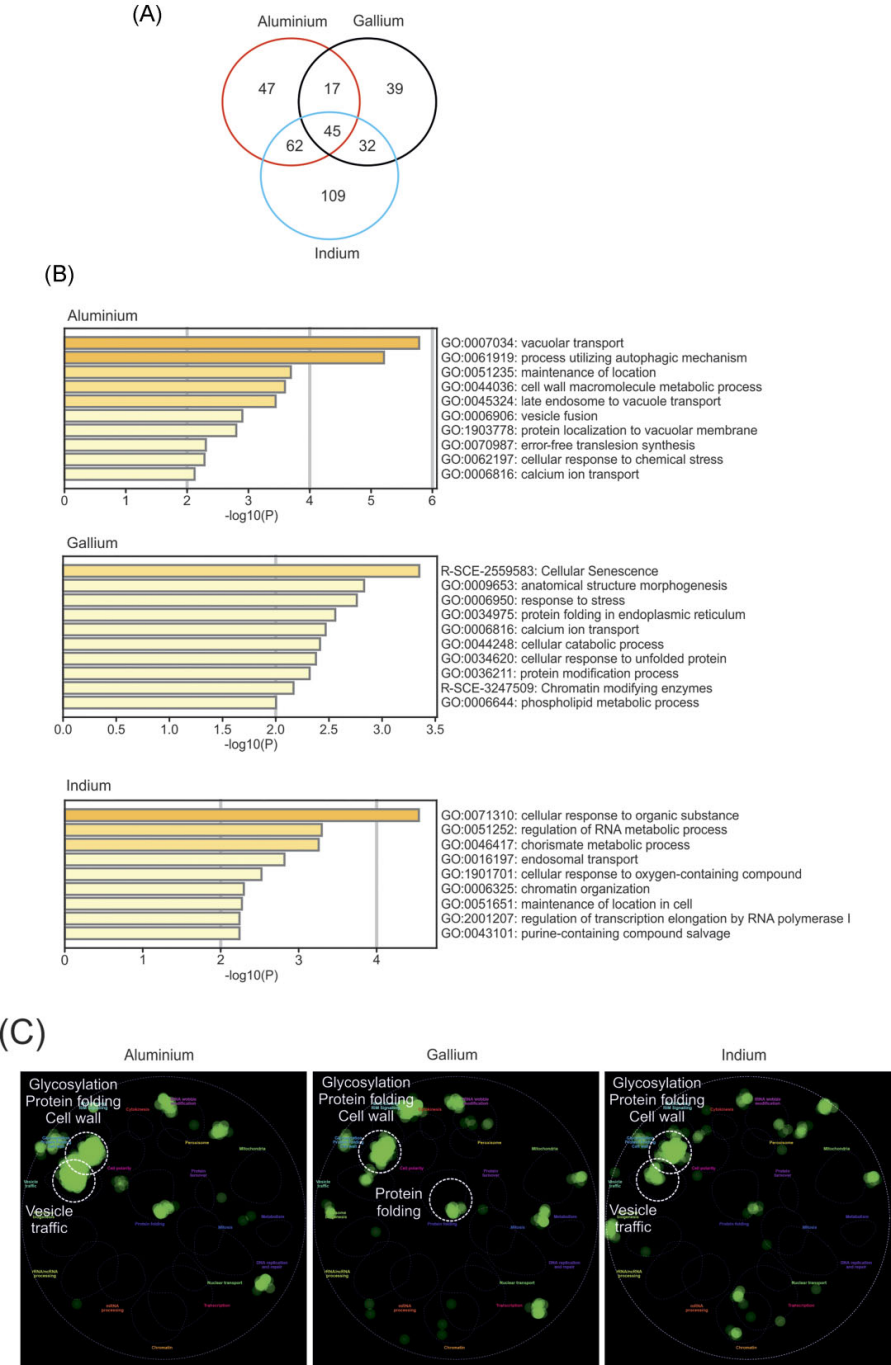
Characterization of the genes that confer Al, Ga, and In resistance. (**A)** The Venn diagram shows the total number and the overlap between the identified genes that confer resistance to Al, Ga, and In. (**B)** Bar plots of top enriched GO-terms in the sets of Al, Ga, and In resistance genes using Metascape.^36^ The colour scale represents statistical significance. (**C)** The hits were mapped onto the global yeast genetic interaction network and visualized using CellMap.^39,40^

We noted that there was a highly significant overlap between the gene-sets conferring resistance to the three metals tested (Fig. 3A): the overlap was 26% (62 of 242 genes) between Al and Ga (*P *< 10^−55^), 25% (77 of 304 genes) between Ga and In (*P *< 10^−64^), and 34% (107 of 312 genes) between Al and In (*P *< 10^−97^). Additionally, there were 45 genes that provided resistance to all three metals. Thus, similar gene-sets appear to protect yeast cells from Al, Ga, and In toxicity.

To identify over-represented gene groups among the genes that confer metal resistance, we performed gene ontology (GO) enrichment and functional protein association network analyses using the STRING^38^ and Metascape^36^ databases.

The set of genes that confer Al resistance was enriched for GO-terms associated with vacuolar transport, autophagy, cell wall macromolecule metabolic process, vesicle-mediated transport, cellular response to chemical stress, and Ca ion transport (Fig. 3B). Additionally, the Al resistant gene products form a highly connected network with significantly more protein–protein interactions among themselves than expected by chance (*P *= 9.9 × 10^−5^) with functions in vesicle-mediated transport, vacuolar transport, and autophagy (Fig. S3).

The genes that confer Ga resistance were enriched for GO-terms associated with cellular senescence, response to unfolded protein and protein folding in the ER, Ca ion transport, chromatin modifying enzymes, and phospholipid metabolic processes (Fig. 3B). Moreover, the Ga resistant gene products form a connected network with more protein–protein interactions among themselves than expected by chance (*P *= 0.006) with functions in the ER and Golgi, and Ca homeostasis (Fig. S4).

The set of genes that confer In resistance was enriched for GO-terms associated with cellular response to organic substance, regulation of RNA metabolic processes, chorismate metabolic process, endosomal transport, chromatin organization, and regulation of transcription elongation by RNA Pol I (Fig. 3B). The protein–protein interaction network of the In resistant gene-set was also enriched for functions associated with the ER and Golgi (Fig. S5).

We next mapped the genes that confer metal resistance onto the global yeast genetic interaction network.^39,40^ This analysis showed that the resistance genes are clustered in distinct functions such as glycosylation, protein folding and cell wall (Al, Ga, and In), vesicle traffic (Al, In), and protein folding (Ga) (Fig. 3C).

Thus, cells appear to protect themselves from Al-, Ga- and In-mediated toxicity through both shared and distinct mechanisms. The overlap between genes and cellular processes that confer resistance to the three metals is probably a consequence of Al, Ga, and In sharing chemical properties and, therefore, affecting cells in similar ways.

### Comparing Al-, Ga-, and In- resistance genes to genes conferring resistance to other metals

**Fig. 4:**
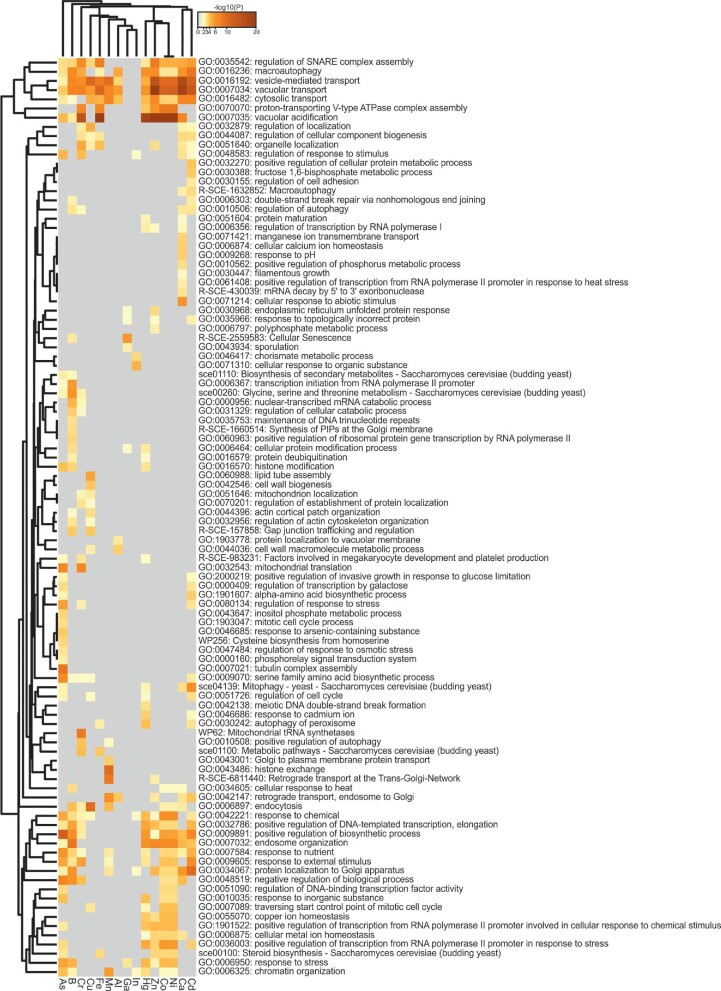
Comparing Al, Ga, and In resistance genes to genes conferring resistance to other metals. Heatmap showing the top enrichment clusters for metal resistance genes. The colour scale represents statistical significance and grey indicates a lack of significance.

We next compared the sets of Al, Ga, and In resistance genes identified in our study to sets of yeast genes that confer resistance to high concentrations of metals from other groups in the periodic table, including arsenite (As(III)), cadmium (Cd),^48^ Ca,^51^ nickel (Ni), cobalt (Co), zinc (Zn), mercury (Hg), Fe,^52^ manganese (Mn),^53^ copper (Cu), chromium (Cr),^54^ and boric acid (B).^55^ The Al, Ga, and In resistance genes clustered close together (Fig. 4), again suggesting similar effects of these group 13 metals on cells. The Al, Ga, and In resistance genes clustered closer to Mn, Fe, and Cu resistance genes than to Cd, Ca, Ni, Co, Zn, Hg, As, and B resistance genes (Fig. 4). Thus, the effects of Al, Ga, and In on yeast cell proliferation may share some common features with Mn, Fe, and Cu. The GO-terms associated with macroautophagy (GO:0016236), vacuolar transport (GO:0007034) and vesicle-mediated transport (GO:0016192), enriched for the Al resistance genes, were also enriched for all other metals except for Ga and In. Thus, these processes appear important for general metal resistance. The GO terms ER unfolded protein response (GO:0030968) and response to topologically incorrect protein (GO:0035966) were enriched for Ga, Cd, and Zn resistance genes, suggesting that these metals may cause ER stress as shown for Cd^56,57^ and Zn,^58^ or that protection against these metals depend on a functional ER. The GO-term chorismate metabolic process (GO:0046417) was enriched exclusively for In resistance genes. Chorismate is the common precursor of aromatic amino acids (phenylalanine, tyrosine, and tryptophan), but is also used for synthesis of other metabolites, including some siderophores.^59^ Thus, chorismate biosynthesis appears specifically important for conferring In resistance.

Although our analyses indicate that some functions are required for resistance to many metals, most resistance genes fall into distinct functional clusters (Fig. 4), in accordance with previous observations.^30,54^ Similar findings were made when comparing our sets of Al, Ga, and In resistance genes to yeast genes conferring resistance to lanthanides^60^: Most enriched GO-terms were metal specific (Fig. S6), although some GO-terms were common for several metals, including Ca ion transport (GO:0006816), which was enriched for Al, Ga, and the lanthanides ytterbium (Yr) and erbium (Er), endosomal transport (GO:0016197), which was enriched for Al, In, and the lanthanides thulium (Tm), terbium (Tb), gadolinium (Gd), dysprosium (Dy), holmium (Ho), and europium (Eu). We speculate that small differences in the physicochemical properties of individual metals could result in different biological targets and cellular responses.

### Calcium homeostasis may be important to protect yeast cells from Al, Ga, and In toxicity

**Fig. 5:**
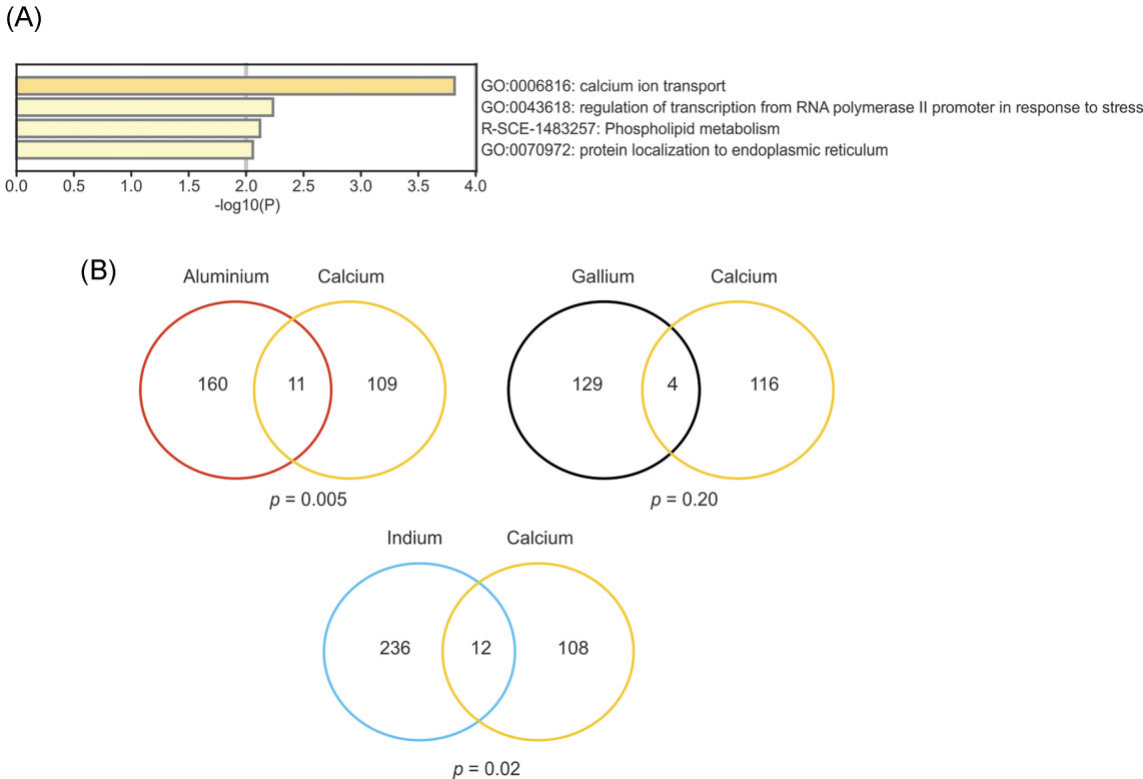
Calcium homeostasis may protect cells from Al, Ga, and In toxicity. (**A)** Bar plot of top enriched GO-terms in the set of 45 common genes that confer resistance to Al, Ga, and In using Metascape. The colour scale represents statistical significance. (**B)** The Venn diagrams show the overlap between the genes that confer resistance to Al, Ga, and In identified in this screen and a set of 120 genes required for Ca resistance.^51^

The common gene-set of 45 genes conferring resistance to all of Al, Ga, and In (Fig. 3A) was enriched for GO-terms associated with Ca ion transport, regulation of transcription, phospholipid metabolism, and protein localization to the ER (Fig. 5A). The proteins encoded by this gene-set form a highly connected network with significantly more protein–protein interactions among themselves than expected by chance (*P *= 0.0016), and several of the common metal-resistance genes encode functions related to Ca transport and the calcineurin complex (Fig. S7). The common gene products include Pmr1 that pumps cytosolic Ca into the ER, Ecm7, and Cch1 that are defective in Ca uptake, Cnb1 encoding the regulatory subunit of calcineurin, and Rcn2, a regulator of calcineurin.^61^ Some of the mutants that lack a resistance gene found in our screen were previously shown to accumulate high levels of intracellular Ca during Ca stress^51^, including *swi4Δ* (Al, Ga, In sensitive), *gph1Δ* (Al, Ga sensitive), *ccz1Δ* (Al, In sensitive), *tps1Δ* (In sensitive), and *csg2Δ* (In sensitive).

To assess whether the Al-, Ga-, and In- sensitive mutants are also sensitive to high Ca concentrations, we compared our gene sets to a set of 120 genes that confer Ca resistance.^51^ We found a significant overlap between genes conferring Al and Ca resistance (11 genes, *P *= 0.005) and between genes conferring In and Ca resistance (12 genes, *P *= 0.02) (Fig. 5B). In contrast, the overlap between genes conferring Ga and Ca resistance was insignificant (4 genes, *P *= 0.20) (Fig. 5B). Altogether, these data suggest that appropriate Ca homeostasis may be an important aspect for cellular protection against Al, Ga, and In toxicity. However, we noted that the genes conferring Ca resistance did not cluster close to the Al, Ga, and In resistance genes (Fig. 4). Thus, the effects of Al, Ga, and In on yeast cells are largely different from those caused by high Ca concentrations. Nevertheless, our finding that Ca homeostasis may be implicated in Al, Ga, and In resistance is in line with previous studies demonstrating that Al disrupts Ca homeostasis and affects Ca signalling in plant cells.^62^

### Disruption of the ER stress sensor Ire1 and the transcription factor Hac1 of the unfolded protein response results in Ga and In sensitivity

**Fig. 6:**
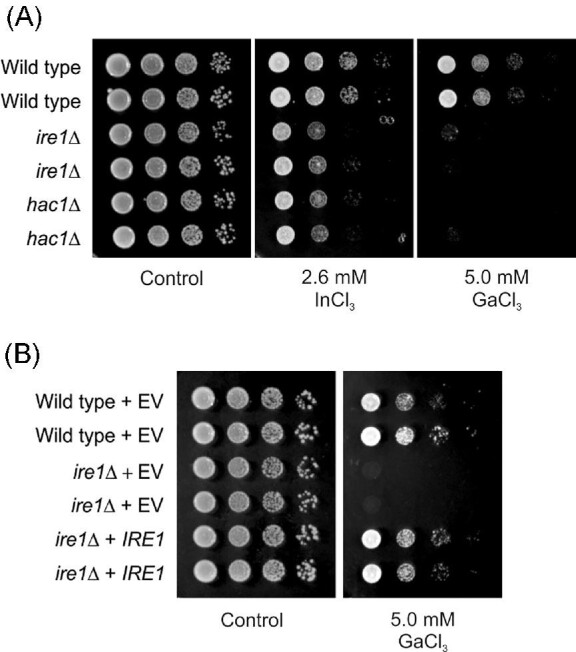
Ire1 and Hac1 protect yeast cells from In and Ga toxicity. (**A)** The indicated strains (BY4741 strain background) were pre-cultured in synthetic minimal medium and 10-fold serial dilutions starting with an OD at 600 nm of 1 were placed on synthetic minimal medium plates containing the indicated concentrations of metal salts. The plates were incubated at 30°C for 3 days. Representative images are shown from at least two independent experiments. (**B)** Growth of wild type and *ire1Δ* cells carrying either an empty plasmid (EV) or a centromeric plasmid carrying the *IRE1* gene behind its endogenous promoter were grown as above.

As mentioned above, the Ga resistance genes were enriched for functions in the ER (Figs. 3B, 4, and S4). The ER has many important roles in the cell, including protein synthesis and folding, lipid biogenesis, and Ca metabolism.^63^ The Ga resistance genes encoding functions related to protein folding in the ER included Ire1 (ER stress sensor), Hac1 (transcription factor of the ER unfolded protein response), Mpd2, Emc1, Emc5, Emc6 (protein folding in the ER), Dfm1, Der1 (ER-associated protein degradation), Erp1, and Cwh41 (protein quality control in the ER). The Ga resistance genes also encoded ER proteins with functions in lipid homeostasis (Ldh1, Sct1, Slc1) and the aforementioned proteins in Ca homeostasis. Impairment of ER functions is sensed, at least in part, by the ER stress sensor kinase Ire1 that, in turn, activates the so-called ER stress response or the unfolded protein response (UPR) via splicing of the *HAC1* mRNA encoding the transcription factor Hac1. Ire1 can be activated by the accumulation of unfolded proteins in the ER and by conditions that perturb lipid membrane homeostasis.^64^ We confirmed that the *ire1Δ* and *hac1Δ* mutants were highly Ga sensitive (Fig. 6A). Additionally, the *ire1Δ* and *hac1Δ* cells were also In sensitive (Fig. 6A). We validated the role of Ire1 in protecting cells against Ga toxicity: a plasmid containing *IRE1* behind its native promoter fully complemented the Ga sensitivity of *ire1Δ* cells (Fig. 6B). Thus, Ire1 and Hac1 functions are important for yeast Ga and In resistance.

Several metals have been shown to induce Hac1 splicing in yeast, including Cd,^56,57^ platinum (Pt),^65^ Co^66^, and Zn.^58^ A study in yeast suggested that Cd activates the Ire1 sensor by perturbing protein folding in the ER^57^, whereas another study suggested that Cd does not inhibit disulphide bond formation in the ER but acts by perturbing Ca metabolism.^56^ Nevertheless, both studies demonstrated that yeast cells lacking either *IRE1* or *HAC1* were Cd sensitive.^56,57^ In may also induce ER stress as shown in zebrafish (*Danio rerio*).^20^ Nonetheless, the mechanisms by which Ga and In affect the ER and induce ER stress remain to be elucidated.

### Human orthologues of Al, Ga, and In resistance genes associated with disease

The knowledge gained from chemical-genomic studies in yeast may be relevant for identifying human health threats since many chemicals target fundamental processes that are evolutionarily conserved. Therefore, we searched the metal resistance genes identified in this study for human orthologues associated with disease using the OMIM database.^41^ At least one human orthologue was found for 80 out of 171 Al resistance genes (47%) of which 35 (20%) are involved in human disease, for 63 out of 133 Ga resistance genes (47%) of which 25 (19%) are involved in human disease, and for 112 out of 248 In resistance genes (45%) of which 38 (15%) are involved in human disease (Table S4). Thus, similar genes may be involved in protecting yeast and humans from toxicity and pathogenicity caused by Al, Ga, and In. This gene set may suggest routes for targeted experimentation and treatment in higher models.

## Conclusions

This study has shed novel light on the chemistry and biology of three group 13 metal elements. Chemical speciation calculations and experimental analyses indicated that Al, Ga, and In form metal-phosphate precipitates in the yeast synthetic complete culture medium. Despite the solubility issues, our study generated meaningful biological data and we identified shared as well as metal-specific defence functions that protect yeast cells against Al, Ga, and In toxicity. While these functions protect yeast from toxicity, dedicated experiments are required to identify metal toxicity targets and to address the mechanisms by which these metals cause toxicity. Several of the identified genes are conserved in humans and are implicated in human disease. The findings from this chemical-genetic profiling screen may provide a basis for further investigations into toxicity and resistance mechanisms in yeast, plants, and humans.

## Supplementary Material

mfad032_Supplemental_Files

## Data Availability

All the data described are available in the article and in its online supplementary material.
