# Supplementary material for: Chemical-genomic profiling identifies genes that protect yeast from aluminium, gallium, and indium toxicity
Source: Metallomics. 2023 May 16;15(6):mfad032. doi: 10.1093/mtomcs/mfad032 (PMC10233895; doi:10.1093/mtomcs/mfad032)
Supplement: mfad032_Supplemental_Files [file mfad032_supplemental_files.zip › Suppl_data_Schulze_R1.pdf]

Supplementary data for:

Chemical-genomic profiling identifies genes that protect yeast from aluminium, gallium, and indium toxicity.

Yves Schulze, Payam Ghiaci, Liqian Zhao, Marc Biver, Jonas Warringer, Montserrat Filella and Markus J. Tamás

Material included:

Supplementary Tables S1 and S2

Supplementary Figures S1 to S7

Supplementary References

Table S1. Input concentrations in speciation calculations (1 mM concentrations for Al, Ga, and In). The charges are omitted for simplicity.

	Concentration [mM]
Cations	
K	7.35
Mg	4.15
Na	1.71
Ca	0.901
Mn	$2.65x10^{-3}$
Fe	$1.23x10^{-3}$
Cu	$2.51x10^{-4}$
NH_4	75.7
Zn	$2.48x10^{-3}$
Anions	
MoO_4	$9.71x10^{-4}$
I	$6.02x10^{-4}$
H_3BO_3	$8.09x10^{-3}$
H_2PO_4	7.35
SO_4	42.0
Cl	3.52

Table S2. Species distribution for 1 mM aluminium, gallium, and indium at pH 4.3 and 25°C in culture medium (main inorganic components only). Supersaturated species correspond to solid species with a solubility index (*SI*) > 0.

Element	Supersaturated species (log*SI* in brackets)	Distribution dissolved species
Aluminium	$AlPO_4.2H_2O$(s) (1.1)<br>$Al_5H_6K_3(PO_4)_8(H_2O)_{18}$(s) (2.0)<br>$Al(OH)SO_4(H_2O)_5$(s) (2.9)	$Al_3(PO_4)_3$ 77%<br>$Al_2(PO_4)_2$ 20%<br>$AlH_3(PO_4)_2$ 2%<br>$AlPO_4$ 1%
Gallium	GaOOH(s) (3.0)<br>$GaPO_4$(s) (3.6)<br>Ga_2O_3(s) (6.7)	$GaHPO_4^+$ 93%<br>$Ga(OH)_3$ 3%<br>$Ga(OH)_2^+$ 2%<br>$Ga(SO_4)_2^-$ 1%
Indium	In_2O_3(s) (0.34)<br>InOOH(s) (1.8)<br>$In(OH)_3$(s) (2.3)<br>$InPO_4$(s) (5.7)	$In(OH)_2^+$ 28%<br>$In(SO_4)_2^-$ 23%<br>$InClOH^+$ 22%<br>$In(OH)^{2+}$ 15%<br>$In(SO_4)^+$ 12%

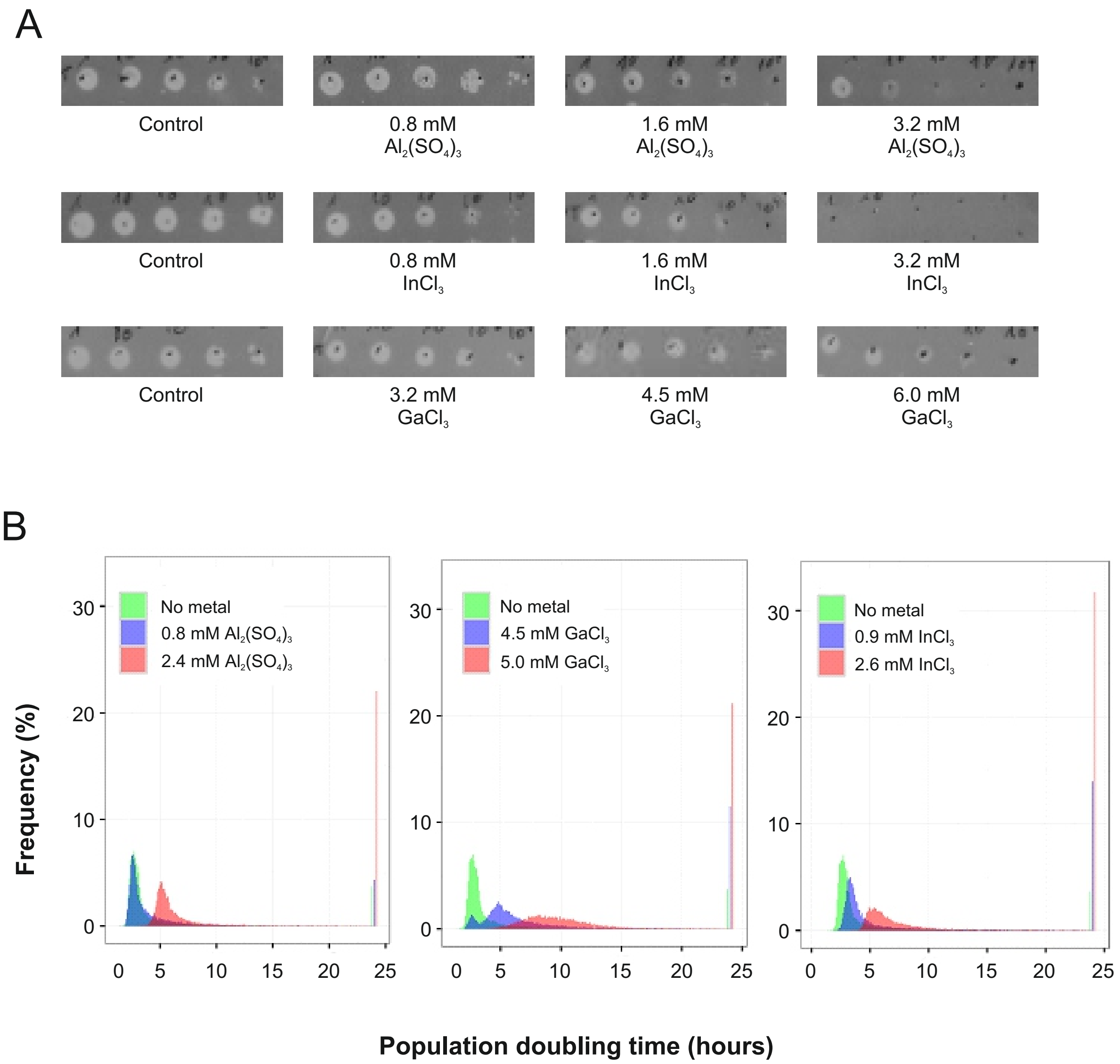

Figure S1. A) Wild type *S. cerevisiae* cells (BY4743 strain background) were pre-cultured in synthetic minimal medium and 10-fold serial dilutions starting with an OD at 600 nm of 1 were placed on synthetic minimal medium plates containing the indicated concentrations of metal salts. The plates were incubated at 30°C for 3 days. Representative images are shown from at least two independent experiments.
B) Absolute growth rate distribution of the yeast deletion collection. Percentage (y-axis) of the distribution of the population doubling time in hour units (x-axis) in medium without metal (green) and in metal containing medium (blue: lower concentration, red: higher concentration) for the whole yeast collection of 4308 single knock-out mutants. Non-growers, with a population doubling time which was ≥ 24 h, were all given a value of 24 h (bars at the right in each plot). Metal concentrations used: Al 0.8 mM and 2.4 mM, Ga 4.5 mM and 5 mM, In 0.9 mM and 2.6 mM. For each metal, the difference is significant (Wilcoxon test, $p<2.2\times10^{-16}$) between no metal medium and lower metal concentration medium and between higher metal concentration and lower metal concentration medium.

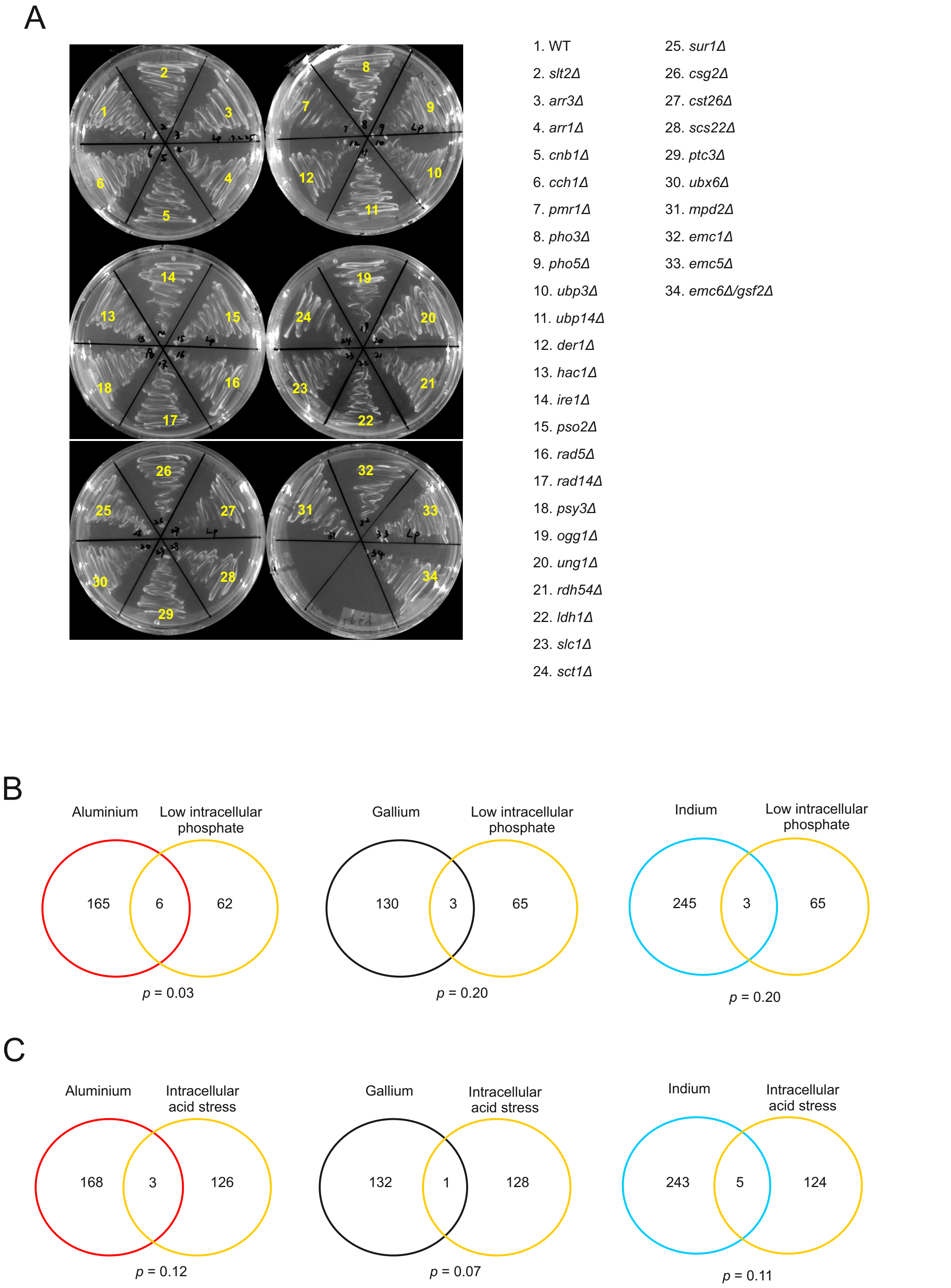

Figure S2. A) The indicated strains (BY4741 strain background) were pre-cultured in synthetic minimal medium and spread on low phosphate synthetic medium containing 64 mg/L KH_2PO_4. The plates were incubated at 30°C for 3 days. Representative images are shown from at least two independent experiments.
B, C) The Venn diagrams show the overlap between the Al, Ga, and In resistance genes and a set of 68 mutants that have low intracellular phosphate levels when grown in rich YPD culture medium [1] **(B)** and a set of 129 mutants that are sensitive to intracellular acid stress[2] **(C)**.

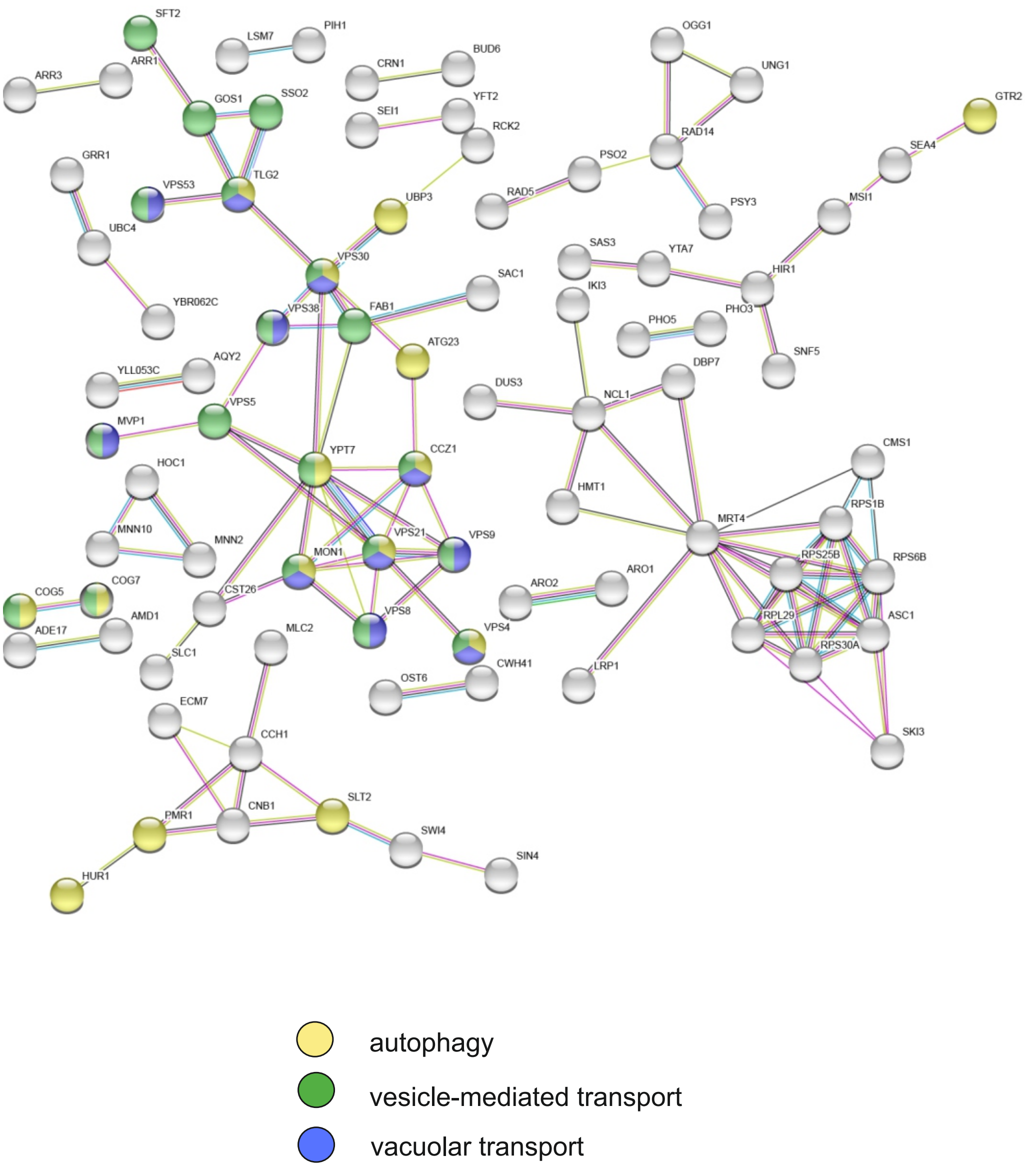

Figure S3. Protein-protein interaction networks among the 171 gene-products that confer Al resistance. Protein-protein interaction networks were constructed and visualized using the STRING database [3, 4] (evidence view, confidence score set to 0.7 (high confidence)). Only connected proteins are shown.

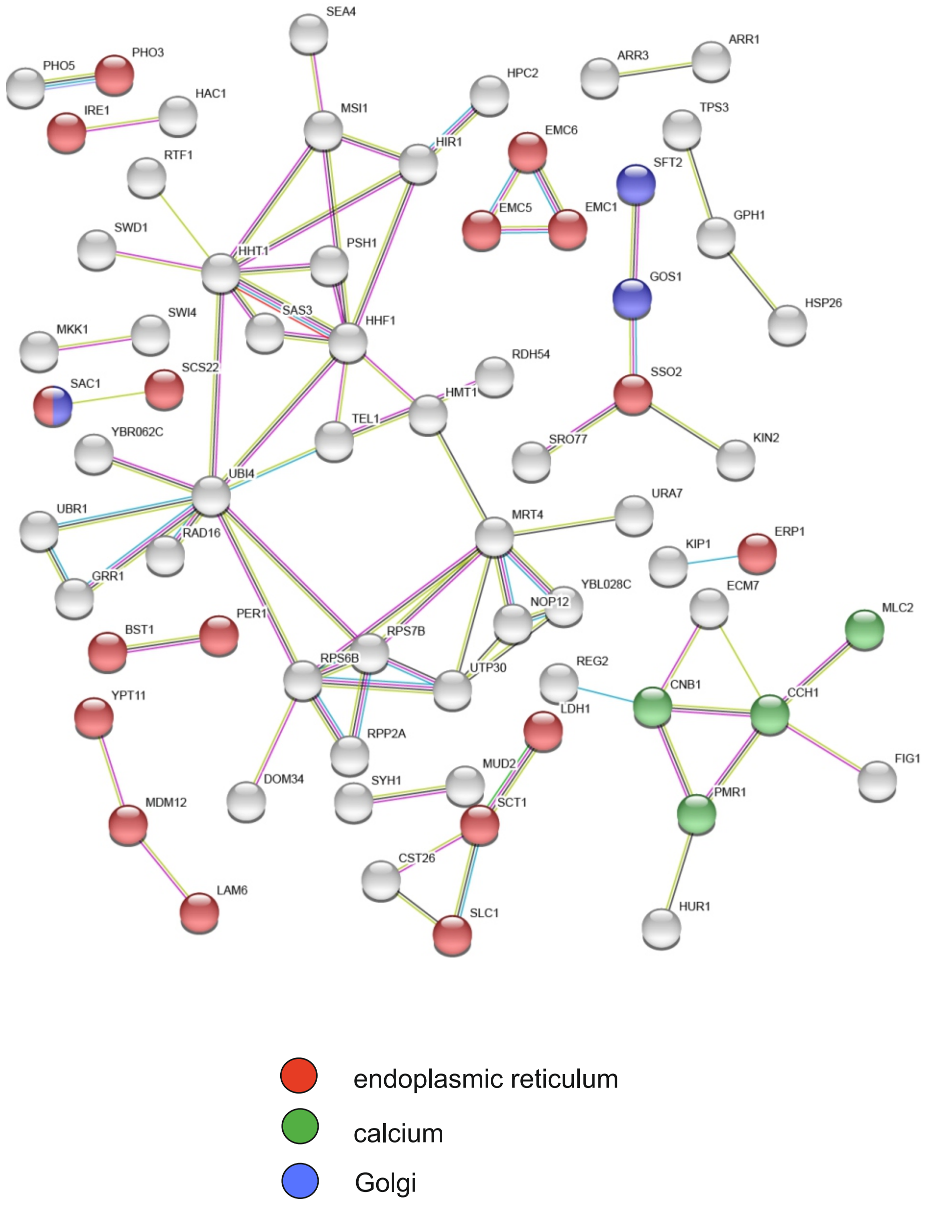

Figure S4. Protein-protein interaction networks among the 133 gene-products that confer Ga resistance. Protein-protein interaction networks were constructed and visualized using the STRING database [3, 4] (evidence view, confidence score set to 0.7 (high confidence)). Only connected proteins are shown.

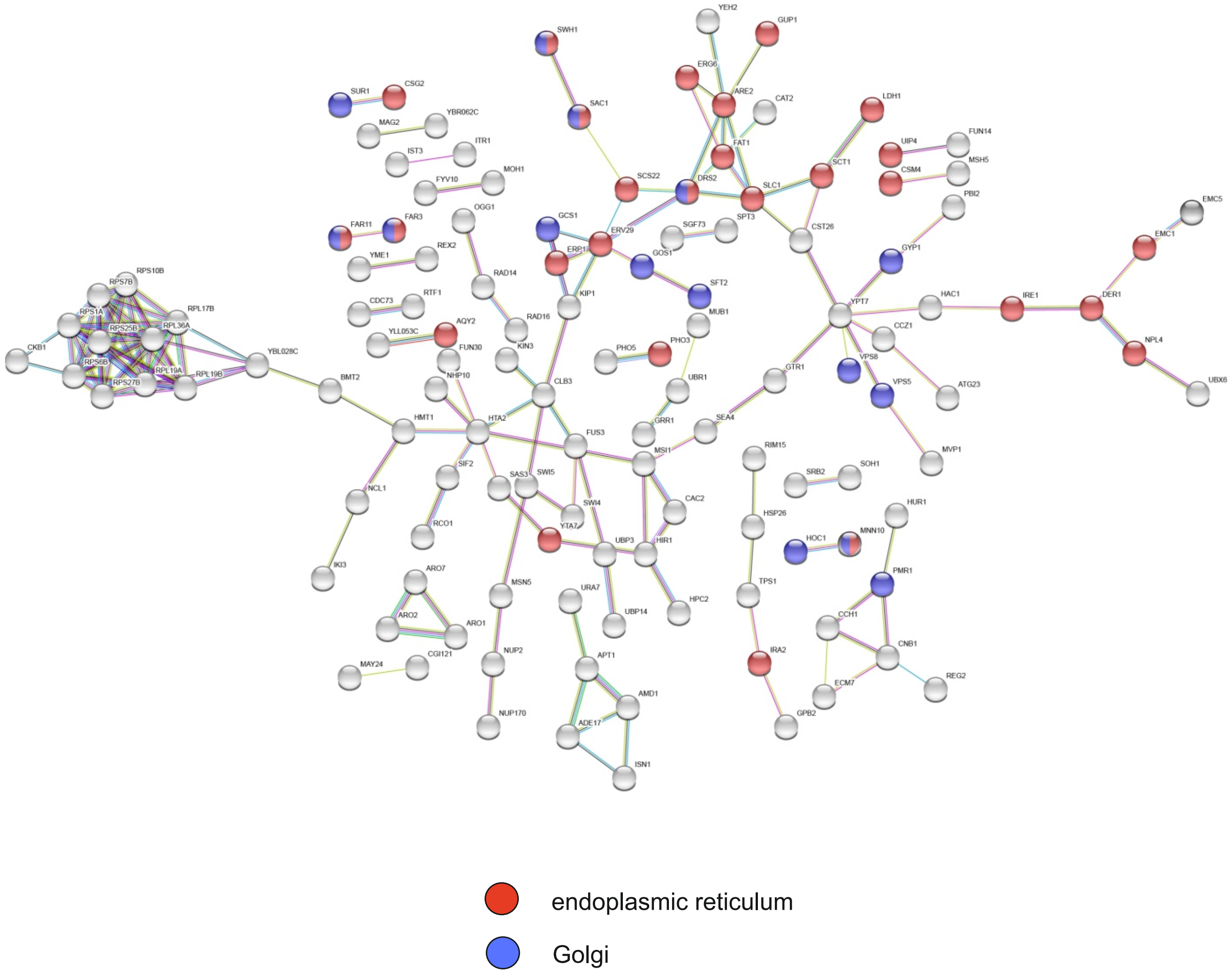

Figure S5. Protein-protein interaction networks among the 248 gene-products that confer In resistance. Protein-protein interaction networks were constructed and visualized using the STRING database [3, 4] (evidence view, confidence score set to 0.7 (high confidence)). Only connected proteins are shown.

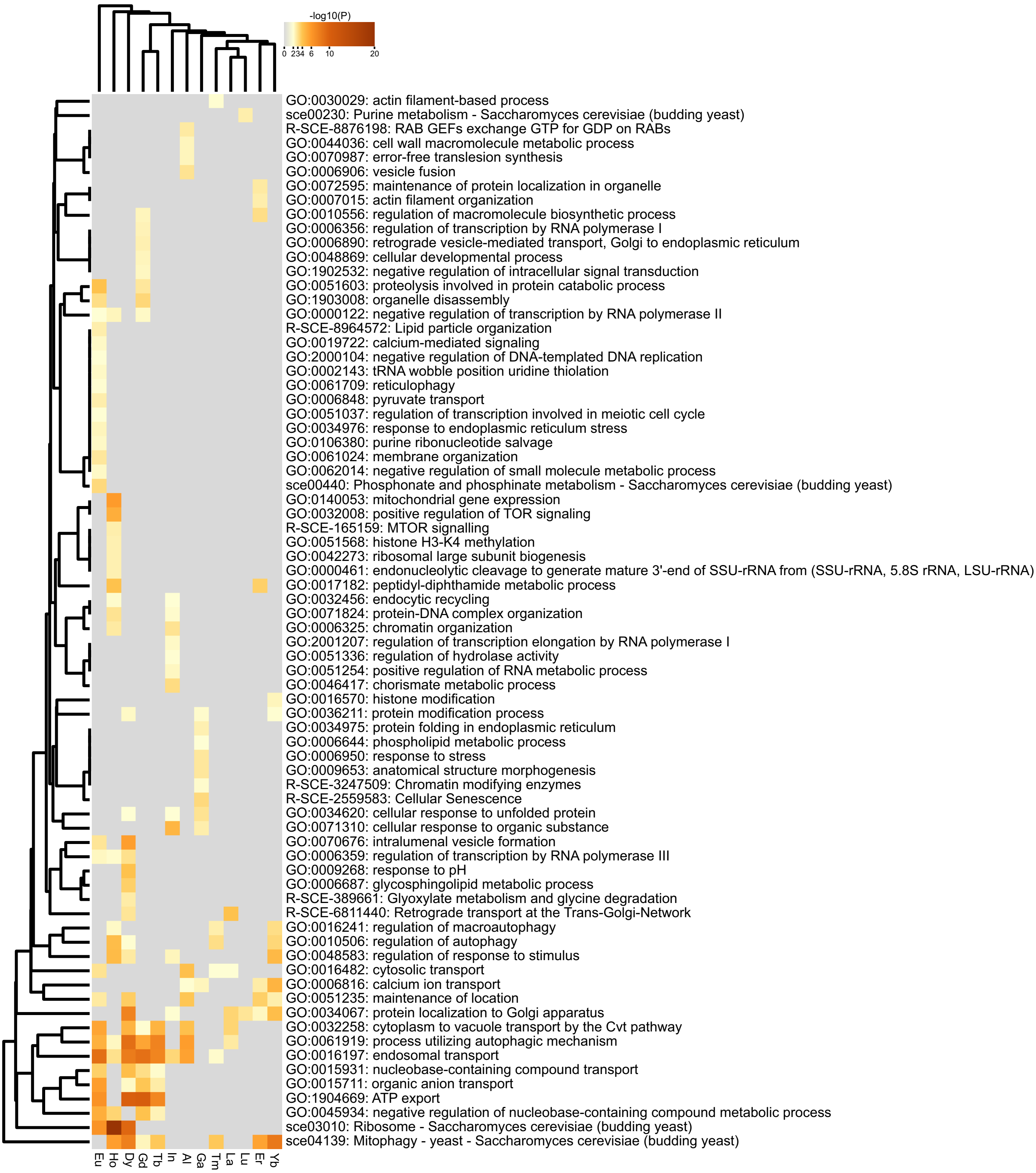

Figure S6. Comparing Al, Ga, and In resistance genes to genes conferring resistance to lanthanides [5]. The heatmap shows the top enrichment clusters for metal resistance genes. The colour scale represents statistical significance and grey indicates a lack of significance.

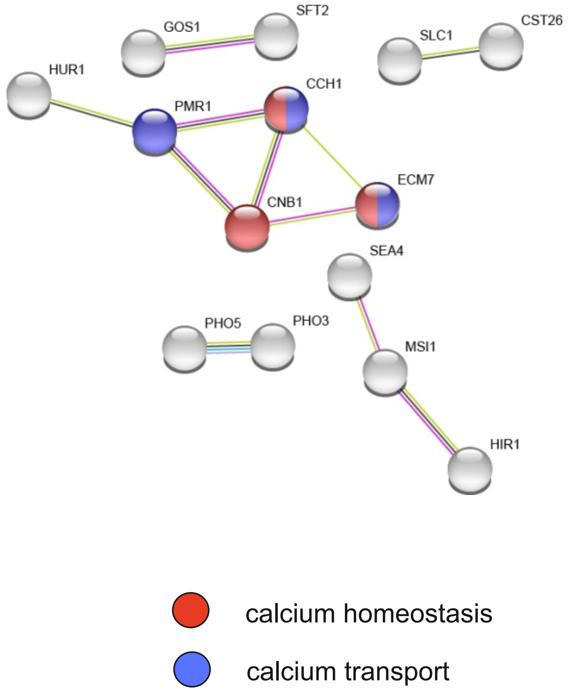

Figure S7. Protein-protein interaction networks among the 45 gene-products that confer resistance to Al, Ga, and In. Protein-protein interaction networks were constructed and visualized using the STRING database [3, 4] (evidence view, confidence score set to 0.7 (high confidence)). Only connected proteins are shown.

Supplementary References

1. Eide DJ, Clark S, Nair TM *et al.* Characterization of the yeast ionome: a genome-wide analysis of nutrient mineral and trace element homeostasis in *Saccharomyces cerevisiae*. *Genome Biol* 2005;**6**(9):R77. doi: 10.1186/gb-2005-6-9-r77
2. Shin JJ, Aftab Q, Austin P *et al.* Systematic identification of genes involved in metabolic acid stress resistance in yeast and their potential as cancer targets. *Disease models & mechanisms* 2016;**9**(9):1039-49. doi: 10.1242/dmm.023374
3. Snel B, Lehmann G, Bork P *et al.* STRING: a web-server to retrieve and display the repeatedly occurring neighbourhood of a gene. *Nucleic Acids Res* 2000;**28**(18):3442-4
4. Szklarczyk D, Gable AL, Lyon D *et al.* STRING v11: protein-protein association networks with increased coverage, supporting functional discovery in genome-wide experimental datasets. *Nucleic Acids Res* 2019;**47**(D1):D607-D13. doi: 10.1093/nar/gky1131
5. Pallares RM, Faulkner D, An DD *et al.* Genome-wide toxicogenomic study of the lanthanides sheds light on the selective toxicity mechanisms associated with critical materials. *Proc Natl Acad Sci U S A* 2021;**118**(18). doi: 10.1073/pnas.2025952118
